# Understanding the Experience and Impacts of Brain Fog in Chronic Pain: A Scoping Review

**DOI:** 10.1080/24740527.2023.2217865

**Published:** 2023-07-10

**Authors:** Ronessa Dass, Mohini Kalia, Jocelyn Harris, Tara Packham

**Affiliations:** a School of Rehabilitation Sciences, McMaster University, Hamilton, Ontario, Canada; b Faculty of Sciences, Carleton University, Ottawa, Ontario, Canada

**Keywords:** Chronic pain, brain fog, chronic musculoskeletal pain, cognitive impairment, dyscognition

## Abstract

**Introduction:**

Approximately 15% to 40% of persons with chronic pain as a primary disorder experience brain fog. Prior research has investigated the etiology of “brain fog” in conditions in which pain presents as a key feature (e.g., fibromyalgia). However, it remains understudied in the context of chronic 10 musculoskeletal pain. Following current scoping review guidelines, we obtained stakeholder input from patient and health care professionals (HCPs) to define this phenomenon. Specific aims of this review were to (1) identify factors contributing to brain fog, (2) identify the functional correlates of brain fog and assessments used to measure them, and (3) establish a definition of brain fog that can be employed by researchers and HCPs to advance research and care.

**Methods:**

A scoping review was conducted using recommendations of the Joanna Briggs Institute methodology of scoping reviews and the Levac et al methodology. Embase, Cinahl, PsycINFO, and Medline was searched to identify relevant sources. Findings were verified with patient and healthcare professionals.

**Results:**

We identified four 15 key features of brain fog: perceived variability, subjective cognitive dysfunction, participation limitations, and changes in functional activities. We developed a model of brain fog illustrating the overlapping categories of contributors to brain fog in chronic musculoskeletal pain: (1) neuroanatomical and neurophysiological, (2) mental health/emotional, and (3) environmental/lifestyle.

**Conclusion:**

The results of this scoping review conclude that the inconsistency in research regarding brain fog in 20 chronic musculoskeletal pain is obstructing a clear understanding of the phenomenon and therefore may be impeding persons with chronic pain and brain fog from receiving optimal care.

## RÉSUMÉ

**Introduction:** Environ 15 % à 40 % des personnes souffrant de douleur chronique en tant que trouble primaire ressentent un brouillard cérébral. Des recherches antérieures ont étudié l’étiologie du « brouillard cérébral » dans des affections dans lesquelles la douleur se présente comme une caractéristique clé (par exemple, la fibromyalgie). Cependant, elle reste sous-étudiée dans le contexte de la douleur musculo-squelettiques chronique. Conformément aux lignes directrices actuelles en matière d’examen de la portée, nous avons obtenu les commentaires des parties prenantes provenant de patients et de professionnels de la santé (PS) pour définir ce phénomène. Les objectifs spécifiques de cet examen étaient de (1) déterminer les facteurs contribuant au brouillard cérébral, (2) déterminer les corrélats fonctionnels du brouillard cérébral et les évaluations utilisées pour les mesurer, et (3) établir une définition du brouillard cérébral qui peut être utilisée par les chercheurs et les professionnels de la santé pour faire progresser la recherche et les soins.

**Méthodes:** Un examen de la portée a été mené en utilisant les recommandations de la méthodologie pour les examens de la portée de l’Institut Joanna Briggs et de la méthodologie de Levac et al. Des recherches ont été effectuées dans Embase, Cinahl, PsycINFO et Medline pour réertorier les sources pertinentes. Les résultats ont été vérifiés auprès des patients et des professionnels de la santé.

**Résultats:** Nous avons recensé quatre caractéristiques clés du brouillard cérébral : la variabilité perçue, le dysfonctionnement cognitif subjectif, les limites à la participation et les changements dans les activités fonctionnelles. Nous avons élaboré un modèle de brouillard cérébral illustrant les catégories se de facteurs contributeurs au brouillard cérébral dans le système musculo-squelettique chronique Douleur qui se chevauchent : (1) neuroanatomique et neurophysiologique, (2) santé mentale/émotionnelle, et (3) environnement/mode de vie.

**Conclusion:** Les résultats de cet examen de la portée concluent que l’incohérence de la recherche en ce qui concerne le brouillard cérébral dans la douleur musculo-squelettique chronique ne permet pas d’avoir une compréhension claire du phénomène et peut donc empêcher les personnes souffrant de douleur chronique et de brouillard cérébral de recevoir des soins optimaux.

## Introduction

“Brain fog” is a term used in both social discourses and the literature to describe a subjective phenomenon of perceived cognitive dysfunction. Brain fog is a multifaceted experience associated with numerous conditions where chronic pain is also a key feature. Approximately 15% to 40% of individuals with chronic pain as a primary disorder experience brain fog.^1^ Chronic pain is a life-altering condition that affects one’s physical, cognitive, emotional, and social well-being, and the experience of brain fog may further worsen quality of life.^2^ Seventeen to 29% of individuals with chronic pain may also experience comorbid mental health disorders: it has been posited these mental health changes may worsen their cognitive capacity and increase the likelihood of experiencing brain fog.^3^

Brain fog does not have a widely accepted definition in the scholarly literature and is most often referred to as issues with attention, memory, and thinking.^4^ One prior narrative review has investigated the etiology of “brain fog” in fibromyalgia, autoimmune disorders, and postural tachycardia syndrome; however, brain fog has yet to be studied in the specific context of chronic pain.^4^ Previous studies have drawn associations between brain fog and memory, attention, and executive function.^4^ However, the potential mechanisms of brain fog in chronic musculoskeletal pain are further obscured by unclear relationships to neuroplasticity, central sensitization, and other changes in brain structure and functional connectivity associated with both primary and secondary chronic pain conditions.^5,6^

Because brain fog may originate differently depending on the specific disorder, it is necessary to understand its development in chronic musculoskeletal pain. For example, some disorders, such as lupus, have no defined biomarker for brain fog.^7^ In long COVID, where COVID-19 symptoms persist for 4 weeks or longer, evolving understanding suggests that decreases in oxygen availability impair mitochondrial functioning, leading to perceived cognitive dysfunction and periods of brain fog.^8,9^ In most cases, disorders have several pathologies that may contribute to the experience of brain fog.

Further, symptoms and severity of brain fog may vary depending on the disorder and based on individual differences and activity demands. In chronic fatigue syndrome, patients often describe brain fog as a generalized “exaggerated state of exhaustion”^10(p4)^ However, symptoms may be more specific in some conditions, such as celiac disease, where brain fog is believed to result in slowed executive function.^11^ Similarly, in fibromyalgia, “fibro-fog” is commonly associated with issues in executive function, attention, and memory.^4^ Frequency and severity also differ based on condition. Ninety percent of patients with neuropsychiatric disorders experience brain fog every day,^12^ whereas patients with COVID-19 may experience brain fog temporarily and infrequently.^8^

Though nonprimary sources have addressed causes and treatment for brain fog in chronic musculoskeletal pain,^9,13^ few studies have actively investigated the topic. Additionally, there have been models developed to explain specific cognitive disruptions in pain, such as Legrain and colleagues’ neurocognitive model of attention to pain.^14^ However, brain fog has not been explicitly described.^14^ Thus, the overarching objective of this review is to investigate the scope and nature of the medical literature defining the concept of brain fog and its corresponding perceived cognitive impairments in persons with chronic pain. For the purpose of this article, all future mentions of chronic pain will refer to primary chronic musculoskeletal pain. Research aims included (1) identifying factors contributing to brain fog, (2) identifying the functional correlates of brain fog and assessments used to measure them, and (3) establishing a definition of brain fog that can be employed by researchers and health care professionals (HCPs).

The findings of these aims can support researchers in investigating brain fog in persons with chronic pain, by providing a definition to include it as an outcome in future studies. In turn, this may assist with the development of reliable assessment measures and effective interventions to help persons with chronic pain manage symptoms of brain fog.

## Methods

Scoping reviews are a form of evidence synthesis that focus on identifying the breadth of knowledge and types of sources on a topic; quality of these sources is not evaluated.^15^ This form of review can be useful to map what is known, identify gaps, and consolidate terminology.^16^ We therefore elected to conduct a scoping review, drawing on the recommended methods from the Joanna Briggs Institute methodology for scoping reviews.^15^ This review was also informed by Levac et al.’s methodology^17^ and sought triangulation of the findings and conclusions from health professionals and lived experience partners.

### Search Strategy

**Figure 1.**
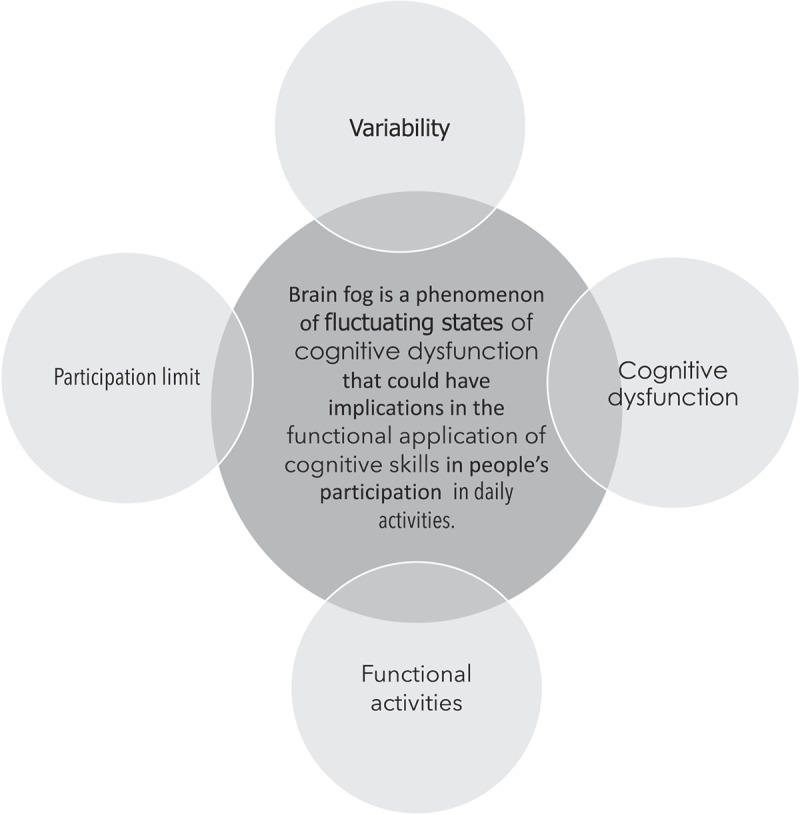
Working definition of brain fog and embedded constructs.

Before beginning any formal search or study screening, a nonexhaustive search of the literature was used to inform a working definition of brain fog. We formulated the following initial definition: “‘Brain fog’ is the term used in the literature to identify a poorly defined phenomenon representing possible variable states of perceived cognitive dysfunction leading to challenges in the day-to-day application of cognitive skills in individuals’ participation in daily activities.” Then, following Levac et al.’s methodology,^17^ this definition was provided to four patient partners and two HCPs specializing in chronic pain. These stakeholders were asked to critically analyze and refine the initial definition. Stakeholders were asked to refine the definition because the initial definition was based upon a rough search of the literature. The authors wanted to ensure that the proposed definition was reflective of patient and HCP perspectives. As a result, we reformulated our working definition for brain fog as “a phenomenon of fluctuating states of perceived cognitive dysfunction that could have implications in the functional application of cognitive skills in people’s participation in daily activities” (see Figure 1 for elaboration). Key features of this definition include possible variability, participation limits, perceived cognitive dysfunction, and functional activities, as highlighted in Figure 1.

The formal search strategy was developed along alongside a librarian and was translated into four electronic health databases: MEDLINE, EMBASE, CINAHL, and PsycINFO. Twenty-seven keywords were identified related to the two main subject areas: (1) chronic pain and (2) brain fog or cognitive dysfunction. Keywords were identified from the literature and patient perspectives. Because our initial nonsystematic search of the literature suggested that brain fog was not explicitly referenced or used as a keyword in studies, a comprehensive list of cognition terms was combined with dysfunction terms (see Appendix A for details). Keywords were combined through Boolean logic terms “AND” and “OR.” Searches were limited to items published by June 6, 2020, when the draft protocol was posted to osf.org (https://osf.io/svr7t) and was updated July 18, 2022. References were exported to the citation manager Mendeley^16^ for deduplication and then to Covidence^18^ to facilitate and track the review process.

### Eligibility Criteria

The same eligibility criteria were used for both abstract and full-text screening.

All selected studies met the following inclusion criteria: (1) sources with participants diagnosed with chronic pain as their primary diagnosis and who are experiencing brain fog or symptoms concordant with our working definition, (2) sources written in English, (3) peer-reviewed primary data sources, and (4) either peer-reviewed or non-peer-reviewed secondary data sources, including reviews. As recommended by the Joanna Briggs Institute, secondary data (e.g., systematic and meta-analytical reviews) are useful sources of information in scoping reviews because they assist in providing a comprehensive understanding of a broader area of knowledge.^15^ Studies were excluded if they primarily focused on participants younger than age 18 because youth may have underdeveloped cognitive abilities in comparison to adult populations.^19^ Similarly, studies focusing on adults older than 65 were excluded because cognitive abilities change with age.^19^ Sources describing participants with chronic pain as a symptom of their primary disorder (e.g., irritable bowel syndrome) were excluded because these disorders were not the main focus of the review and may have complicating comorbidities and their own specific variation of the concept of brain fog. Next, we excluded studies concentrating on participants with traumatic brain injuries, neurodevelopmental disorders (e.g., autism spectrum disorder) or other cognition-impairing disorders (e.g., dementia) because these conditions may have unique effects on cognition that may confound the potential effects of pain.^19,20^ Sources that primarily investigated changes in cognition due to medication were excluded. Additionally, sources for which full text could not be obtained were excluded; however, attempts to contact authors were made using e-mail and professional networking sites such as ResearchGate and LinkedIn. Lastly, we excluded gray literature (e.g., blog post, books, scripts, conference abstracts, etc.). We report that this is a deviation from our published protocol, because it was initially stated we would include gray literature such as dissertations and opinion papers. Upon screening, the authors decided to limit the inclusion criteria to peer-reviewed papers only to support the quality of reported results.

Titles and study abstracts were independently screened by two reviewers (R.D. and M.K.). Studies selected for full-text screening were independently analyzed by two reviewers (R.D. and M.K.). Conflicts from all stages were resolved by discussion between all three reviewers (R.D., M.K., and T.P.).

### Data Collection

The study design characteristics extracted included (1) author and year, (2) type of study and study rating as suggested by the Centre for Evidence-Based Medicine,^21^ (3) sample size, (4) type of task and/or treatment,^22^ (5) method of analysis, and (6) professionals involved. Patient demographic information was compiled, including sex, race, pain diagnosis, socioeconomic status, and age. Further, available detailed information on brain fog was extracted: (a) any definition of brain fog, (b) cognitive ability measurements, (c) sleep measurements, and (d) quality of life measurements and effects. These categories were informed by the constructs we identified within our working definition of brain fog (see Figure 1). We also extracted information to inform future studies including proposed next steps for research, described evidence gaps, and study implications and limitations.

### Study Selection

**Figure 2.**
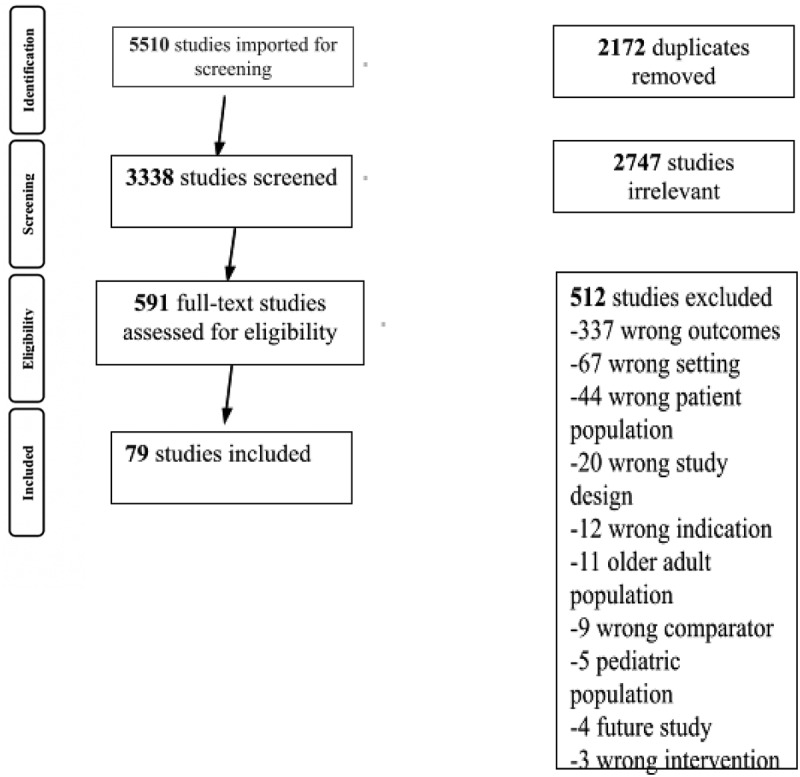
Prisma diagram.

The electronic search yielded 4869 abstracts for screening after removal of duplicates (see Figure 2 for PRISMA [Preferred Reporting Items for Systematic Reviews and Meta-Analyses] diagram). A final total of 79 papers were included for data extraction.

### Thematic Summary

The thematic summary was conducted through an iterative process, following guidance of Braun and Clark.^23^ After familiarization with the extracted data, two authors (R.D. and M.K.) independently noted patterns and salient themes in the data. The themes were further refined through regular meetings and discussion with the research team for crystallization^23^ until consensus was achieved.

## Results

### Description of Studies

The literature identified relied on quantitative methods with the exception of a sole qualitative paper that described a focus group exploring the experience of exercise in persons with fibromyalgia.^24^ A broad spectrum of empirical study designs was recorded, including three meta-analytical reviews,^25–27^ two systematic reviews,^28,29^ three reviews,^30–32^ and three randomized controlled trials (RCTs)^33–35^; however, the majority were lower levels of evidence such as cross-sectional (*n* = 55) or uncontrolled cohort designs (*n* = 2) exploring some aspect of cognition, attention, judgment, or memory in persons with chronic pain (see Appendix B for a complete account of the designs and foci and additional study information).

### Description of Population

The majority of the studies included female participants with either chronic pain or fibromyalgia. The exception was studies investigating low back pain, which generally consisted of equal numbers of males and females or included comparatively more male subjects.^36–38^ Mean age of chronic pain manifestation ranged from 32.9 to 48.4 years of age. For fibromyalgia, this was slightly later: patients developed symptoms starting at 38.0 to 52.0 years old. Though most studies primarily included White participants, some literature investigated chronic pain symptoms among different ethnicities. Though socioeconomic status and education were not frequently addressed in the collected literature, one study stated that pain symptoms were more common in patients with an annual income of at least $45,000.^39^ Two studies reported that chronic pain symptoms in their samples were more frequent in individuals who had obtained either secondary or university education.^40,41^

### How Is Brain Fog Currently Defined in the Literature?

Of the 79 papers, the majority (*n* = 78) of articles did not explicitly define brain fog (see Appendix B for a summary table for included papers). Eight papers defined fibro-fog as a specific phenomenon in patients diagnosed with fibromyalgia.^27,40,42–47^ Fibro-fog has been broadly described as cognitive impairments in fibromyalgia^27,47,48^; however, the most common features include issues with attention and memory.^45,46^ For example, Gunendi et al.^44^ described fibro-fog as a state of impaired central processing of sensory stimuli characterized by difficulty focusing attention, remembering new information, making decisions, and performing tasks. Glass et al^43^ suggested that it was primarily patients who called problems with memory and concentration fibro-fog but suggested that “dyscognition” was the term used more often in the medical literature.^30,43^ A single study offered brain fog as a synonym for fibro-fog, noting that it represented neuropsychological changes including memory, concentration, and attention deficits.^49^ Williams et al. studied cognitive dysfunction in fibromyalgia with a focus on elucidating the multiple experiences and broad spectrum of patient reports related to dyscognition or fibro-fog, arguing that it was more than memory problems.^50^ Interestingly, the publication listed brain fog as a keyword in addition to fibro-fog; however, the main manuscript failed to use the term brain fog.^50^

Taylor et al. used brain fog in their survey as a plain-language synonym for cognitive dysfunction but did not provide a definition to either survey participants or in their manuscript.^51^ Two additional papers used the term brain fog related to refer to cognitive changes; however, they did not explicitly define it, nor was brain fog the main focus of either article.^43,51^

**Figure 3.**
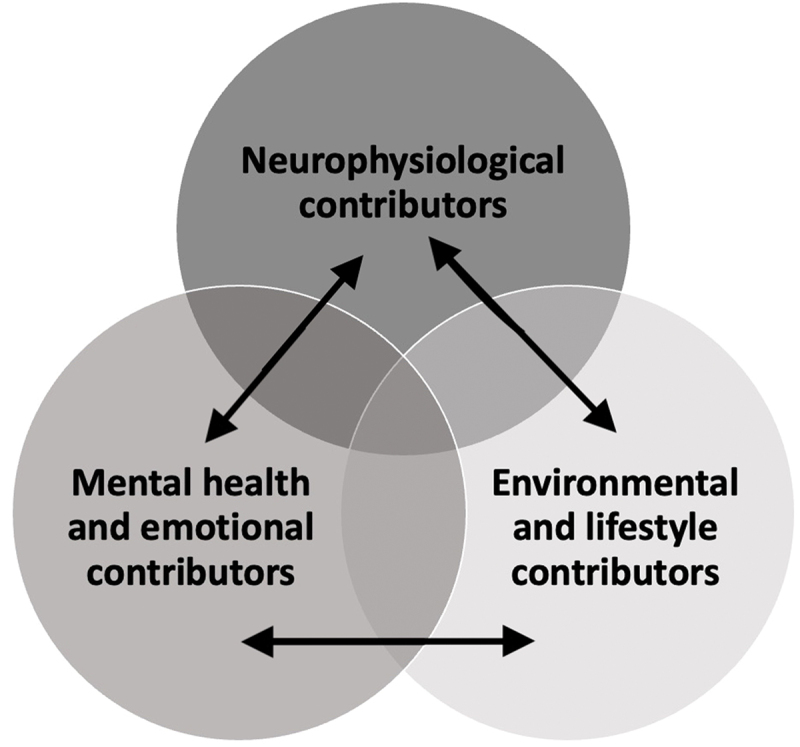
Brainfog contributors model.

From these definitions and descriptions, we identified three overlapping categories to organize factors contributing to brain fog in chronic pain: neurophysiological, mental health and emotional, and environmental/lifestyle factors. This thematic summary is depicted in Figure 2, with all papers corresponding to the brain fog factors (Figure 3a,b).

### Does Brain Fog Vary across Chronic Pain States?

The majority of papers included in this review used homogenous patient samples, with persons having the same diagnosis and at times even came from the same clinic or community.^45^ Few studies directly compared different pain populations; however, some papers did find cognitive differences between pain groups.^52–59^ Koutanji et al. compared pain interference in cognition in individuals with higher or lower levels of chronic pain.^56^ Dick et al. found that persons with fibromyalgia and rheumatoid arthritis had lower cognitive performance in comparison to persons with musculoskeletal pain and pain-free individuals.^53,^ Interestingly, patients with musculoskeletal pain performed similarly to pain-free individuals. Similarly, Castel et al. found that participants with fibromyalgia self-reported more memory complaints than participants with chronic pain.^60^ Further, Coppieters et al. reported that women with chronic whiplash-associated disorders both self-reported more cognitive deficits and performed lower on objective measures of cognition than women with chronic idiopathic neck pain.^61^

## Thematic Summary: Aim #1 Factors Contributing to Brain Fog

The following sections outline a thematic analysis of identified contributors that have been correlated to the subjective experience of brain fog, namely, (1) neurophysiological contributors, (2) mental health and emotional contributors, and (3) environmental lifestyle contributors.

### Neurophysiological Contributors

Thirty-one papers discussing the neurophysiological contributors of brain fog were identified. A 2018 literature review by Mazza et al. noted several studies have found an association between chronic pain and structural changes in the brain.^32^ Several authors posited that pain modulation dysfunction may create symptoms of brain fog, because pain processing takes away from mental resources and therefore decreases the brain’s ability to attend to other cognitive functions.^27,43,62,63^ Hence, increased intensity and chronicity of pain are theorized to increase both objective and self-reported severity of brain fog symptoms.^32,48,61,63–66^ Pain interference, especially when it involves multiple aspects of one’s daily activities, has been found to be predictive of objective and subjective cognitive impairments.^67,68^

Glass et al. stated that pain processing overused resources in the prefrontal cortex (PFC), which decreased cortical inhibition during cognitive assessments.^43^ Similarly, Ren et al. noted that decreased activation of the PFC as well as decreased oxygen-hemoglobin flow may contribute to some cognitive deficits.^66^ Neural network changes have also been reported, including reduced neuronal inhibition, which may indicate that neural signaling between networks is slowed.^69^ Changes in hormone levels were also identified. For example, changes in dopaminergic neurotransmission or hypoperfusion have been documented in the thalamus and insula.^41,49^

### Mental Health and Emotional Contributors

Sixteen papers discussed mental health and emotional contributors to brain fog. Mental health comorbidities and emotional stress are prevalent in persons with chronic pain. Emotional states or conditions, such as anxiety and depression, use an abundance of cognitive resources, potentially overwhelming and prohibiting typical cognitive functioning.^32,41,70,71^

This was demonstrated in a 2021 study by Castel et al. in which, which participants with depression were excluded from the analysis, participants with fibromyalgia and chronic pain had similar levels of sustained attention.^60^ Similarly, Galvez-Sánchez et al. concluded that negative affect, higher levels of anxiety, alexithymia, and pain catastrophizing, as well as lower levels of self-esteem, were all correlated with worse performance on neuropsychological measures.^42^

An RCT using computerized training to aid in the improvement of cognitive impairments in chronic pain found that improved objective and self-reported levels of anxiety, depression, and catastrophizing were associated with improved self-report levels of cognition.^33^ Additionally, Sephton et al. reported excessive cortisol release associated with stress, chronic pain, and the prevalence of mental health comorbidities.^72^ These factors contribute to release of excessive amounts of glucocorticoid hormones, which impairs declarative memory and were thought to contribute to the subjective experience of brain fog.^72^

### Environmental and Lifestyle Contributors

Lifestyle and environmental factors were discussed in six papers. McCracken and Iverson described how stressful life factors can act as a contributor to brain fog.^65^ For example, financial, familial, or work stressors can cause rumination and depressive symptoms that affect cognitive functioning.^65^ Medications to alleviate pain symptoms may also produce side effects that hinder cognition.^65,68,73^ Another aspect of lifestyle that may contribute to brain fog is frequency of sleep. Self-report measures indicate that a lack of sleep, or irregular sleep, has been theorized to hinder cognitive processing and decrease levels of attention, memory, and executive functioning.^41,74–76^ Conversely, two studies identified in the search argued that sleep disturbances have little effect on cognitive performance.^68,75,108^ For more information regarding sleep, please see thematic summary #2.

### Overview of Contributors

As demonstrated above, pain-induced neurophysiological changes, mental health and emotional contributors, as well as environment/lifestyle contributors can act independently to contribute to the experience of brain fog. However, factors may also act synergistically. For example, mental health comorbidities^74^ and high pain intensity^50,63–65^ both use an abundance of cognitive resources, and a combination of the two may lead to cognitive overload. Further, factors can have dual effects, meaning that they influence each other. As an example, the presence of chronic pain has been found to affect brain morphology by reducing gray matter volume in the ventromedial PFC, the insula, and the dorsolateral PFC.^77^ These morphological changes may in turn affect the ability of these regions to engage in their typical cognitive functioning.^77^ Additionally, inadequate sleep may contribute to symptoms of brain fog; however, the occurrence of brain fog could also hinder one’s sleep.^75,78^ The presence of chronic pain could also affect both of these factors.^42,53^ Similarly, continuous stress from life stressors can contribute to brain fog, and the experience of brain fog can also increase one’s stress.^65^ The exact causality of these relationships is unclear.

Figure 3 provides a Venn diagram illustrating the complexity of the contributors of brain fog. The independent circle represents the three primary types of contributors acting independently. The overlapping regions of the Venn diagram demonstrate that contributors may act synergistically. Finally, the arrows illustrate that contributors can have a correlational and bidirectional relationship.

## Thematic Summary: Aim #2 What Functional Correlates of Brain Fog Have Been Described and How Are They Assessed?

Impairments attributed to brain fog were reported by 42 papers and thematically grouped into three main areas: cognitive function, sleep, and quality of life.

### Cognitive Function

Brain fog in chronic pain was reported to affect a wide range of cognitive processes (32 studies). Identified impairments related to brain fog included changes in memory, language, executive function, attention, and global cognition (see Appendix C for a summary of impairments and the tests used to measure them). The tests used to measure the listed impairments were inconsistent across studies, with over 65 different tests being used. Tests were primarily of three types: cognitive screening (e.g., Montreal Cognition Assessment^74^) performance-based (e.g., Iowa Gambling Task^52^), and self-report (e.g., Multiple Ability Self-Report Questionnaire^50^). Measures used were appropriate for the patient population and overall had strong reliability and validity; however, they were mostly general measures of screening (e.g., Weschler Memory Scale and Stroop task). Some studies did use assessments that were more specific and assessed how cognitive impairments intervened with one’s ability to participate in daily activities (e.g., Everyday Memory Questionnaire^79^ and Test of Everyday Attention^53^).

The fact that numerous questionnaires were used across studies is problematic because this makes it difficult to compare cognitive abilities among different study samples and obtain an understanding of how cognition is affected by chronic pain. This statement echoes a 2022 systematic review performed by Zhang et al. that declared the need for a unified method of cognitive evaluation in chronic pain studies.^29^ Further, questionnaires currently used in most studies do not capture the subjective experience of brain fog in persons with chronic pain and its effect on daily living.

### Sleep

Twenty-five studies addressed the relationship between brain fog, chronic pain, and sleep. Sleep deficits have been reported as a direct symptom of fibro-fog.^45,50,51^ Other studies have described it as a probable symptom and have measured it through self-report questionnaires.^27,37,41,43,45,53,56,61,67,68,72,76,78,80–85^ Interestingly, three studies measured sleep disturbances in patients with chronic pain and perceived cognitive dysfunction; however, they did not describe sleep impairments as a symptom of brain fog.^38,53,86^ The bidirectional relationship between brain fog, chronic pain, and sleep has also been documented in the literature.^32^

Two studies theorized that sleep disturbances can exacerbate perceived cognitive dysfunction and that brain fog can contribute to problems with sleep.^75,78^ To make matters even more complex, chronic pain can also contribute to both sleep disturbances and perceived cognitive dysfunction.^75,78^ This relationship has not been confirmed, because other studies did not find that sleep disturbances were correlated with perceived cognitive dysfunction.^14,68,74^

It is important to note that sleep assessments were mostly self-report and are therefore subject to self-report bias; however, no objective measurements were identified. Additionally, most study findings regarding sleep, brain fog, and chronic pain were correlational in nature and did not directly study their relationship.

### Participation in Daily Activities and Quality of Life

Because quality of life is a broad term that may have different meanings depending on the context, this article defined quality of life as pain experiences and beliefs, the interaction between pain and emotional state, and one’s participation in daily activities. McCracken and Iverson used a self-report measure to examine the frequency of cognitive complaints and the resulting challenges these complaints produced in the ability of persons with chronic pain to engage in daily activities.^65^ They found that 23.4% of participants reported forgetfulness, 23.1% minor accidents, 20.5% difficulty with task completion, 18.7% difficulty with attention, and 54% impairments in with at least one cognition function.^65^ Similarly, Iezzi et al. noted that participants often stated they experience difficulty in managing medication, daily tasks, decision making, and social interactions.^82^ Further, Seward et al. found that driving abilities were reduced in participants with higher pain intensity and emotional dysfunction.^38^

Similar to cognitive impairments, the assessments used to measure activity and quality of life in persons with chronic pain experiencing brain fog were inconsistent across studies: we recorded use of 26 different instruments addressing a variety of constructs including general health, mental health, the pain experience, and overall ability to perform daily activities (see Appendix D for papers describing impairments to quality of life and how they were measured). Measures used are commonly used in the chronic pain population and had strong validity; however, it was once again difficult to make comparisons across studies because of the high variability of assessments used. Further, the utility given the context of the assessments’ usage and patients’ understanding of questionnaires is unclear. For example, the Brief Pain Inventory is a self-report measure used to assess one’s pain interference scores. Although we were not able to identify any empirical linkages, we hypothesize that patients may perceive cognitive dysfunction as an example of pain interference, rather than seeing it as something distinct. This uncertainty and lack of distinction may act as a barrier toward our understanding of the unique impact of brain fog in persons with chronic pain.

## Aim #3: Definition

Our working definition (see Figure 1) includes three key elements or constructs of brain fog that were developed within this literature review. The first construct is possible variability in the symptoms, frequency, and severity of brain fog.^50^ This variability was framed as complex in the work of Williams et al. who described multifaceted contributions and drew on patient self-report measures to capture the fluctuations. However, we were unable to identify any longitudinal studies on dyscognition in chronic pain, and thus empiric evidence for within-subject temporal variability is lacking. The heterogeneity seen within groups can not only be attributed to temporal fluctuations in frequency and indeed may be a better representation of the possible variable severity, contributing to the sometimes small differences seen between persons with pain and healthy controls.^69,72,78^ The second construct in the working definition that merits highlighting is participation limitations. Participation limitations refers to the challenges that individuals encounter when performing daily activities. The findings of this review represented participation most clearly in measures of quality of life. This relates to third construct of functional activities. The last construct is perceived cognitive dysfunction, which is an umbrella term accounting for impairments to attention, memory, executive, and overall cognitive function.^43,50,55,69^ After analysis of the literature, we propose that the definition “brain fog is an amorphous subjective phenomenon used in the literature to describe the experience of (1) possible variable states of perceived cognitive dysfunction that may lead to (2) challenges in the (3) functional application of cognitive skills for participation in (4) daily activities” be used in future research investigating brain fog in chronic pain.

## Discussion

The inconsistent and vague use of brain fog seen in the medical literature illustrates the importance of the present scoping review and established definition. Despite the reported importance to persons living with chronic pain conditions,^44^ at present there is no clear or widely accepted definition or diagnostic criteria for the brain fog phenomena in persons with chronic pain. Correspondingly, this review was guided by a consensus definition from clinical and patient stakeholders in an attempt to provide a clear understanding and definition of brain fog. Overall, the model developed from our findings demonstrated that cognitive, biological, and environmental factors may act independently or synergistically to produce symptoms of brain fog. These symptoms primarily appear as impairments to cognition, sleep, and quality of life and may act bidirectionally to contribute to the experience of brain fog (see Figure 3). While sharing the conception of bidirectional influences, our model expands on the neurocognitive model of attention to pain proposed by Legrain et al.^14^ because we have chosen to look more broadly at dyscognition.

Further, the current method of assessment is problematic and prevents a proper understanding of brain fog. Given that studies do not use consistent metrics of brain fog, it is difficult to make meaningful comparisons and to obtain a coherent understanding of individuals’ experiences. Currently, the measurements that are used do not explicitly account for the subjective phenomenon of brain fog and do not capture its multidimensional nature (see Appendixes C and D). Because most assessments are standardized and because there are few qualitative studies investigating brain fog, the real burden of brain fog in patients’ lives is unclear. Further, we excluded any papers primarily focusing on addressing changes in cognition related to medication management; however, we acknowledge the need to understand the independent contributions of brain fog and medication as well as to understand whether the experience of brain fog differs in persons taking pain medications with known cognitive sequelae.

### Is Brain Fog a Synonym or Subtype of Dyscognition?

When conducting this review, we made the assumption that brain fog and dyscognition were similar in nature and thus included both terms in our search. However, our findings demonstrate that the similarities and differences between the two terms are unclear, because they may both be overly or incorrectly used in the medical literature. Glass et al. stated that dyscognition may be the official term for brain fog used in medical literature.^43^ Dyscognition has been described as both self-reported symptoms and objective impairments.^30^ This suggests that brain fog and dyscognition may represent the same phenomenon. However, dyscognition has also been mentioned as a feature of brain fog.^43,50,55,69^ The inconsistent and broad use of the two terms demonstrates the urgent need for the terms to be properly defined. If dyscognition and brain fog are the same experience, then establishing this definition will facilitate comparisons between studies and patient experiences. Conversely, if brain fog is a different (perhaps more specific) experience than dyscognition, a coherent definition will help to distinguish the two phenomena. This can inform the selection of formal assessments and personalized treatment planning.

### Gaps in the Literature

Our review highlights important research gaps. For example, brain fog in chronic pain is a complex disorder that manifests uniquely in each individual; however, little is known about the effects of individual differences and factors. Empirical research investigating these factors may help to provide a better understanding of this perplexing phenomenon. For example, much of the research on chronic pain and brain fog focuses on fibromyalgia, and brain fog in other chronic pain conditions remains understudied.^2^ Research investigating the experiences of other chronic pain conditions is necessary, because brain fog can be initiated or affected uniquely in different conditions. Some studies have found that neuropathic pain produces stronger cognitive impairments than other pain conditions.^5–7,87,88,89^ Additionally, individual factors such as differences in gender, sex, culture, age, socioeconomic status, fatigue, mental health, and personality traits may contribute to the experience of brain fog and explain the variation in prevalence in persons with chronic pain.^8–13,32^ Moreover, the understanding of how these factors interact to produce symptoms of brain fog is unclear. As an example, emotional difficulties (e.g., depressive and anxious symptoms) use cognitive resources, which, when combined with pain, may produce brain fog.^32,60,68,82^ However, emotional difficulties may also be a result of the experience of brain fog. The nature of this interaction should be investigated in future studies and should consider other potential mediators and/or moderators such as pain intensity and sleep in these relationships.^5–7^ The interactive, or additive effect, that medication may have on the relationship between chronic pain and brain fog also requires further investigations, because some studies reported an effect,^87^ whereas others did not.^8,32^

Further, the role of hormones and immune influences in the experience of brain fog are worthy of exploration. However, given the lack of a clear definition of brain fog, it is difficult to compare the experience of brain fog across conditions or pain states, again highlighting the need for a clear definition and robust metrics.

To account for the heterogeneity of brain fog in chronic pain, we echo necessary modifications to study design that have been proposed or illustrated by the existing literature. First, larger sample sizes with diverse pain populations are crucial for future research, because brain fog is a complex disorder that is affected by multiple factors.^35,36,43,61,63,67,77,89–92^ Currently, sample sizes are smaller in nature and more frequently focus on homogenous pain samples. Understanding these factors will facilitate HCPs’ ability to follow a precision medicine approach, which may be the only appropriate method to treat the variability of brain fog in persons with chronic pain. Study designs that are necessary to investigate these factors include longitudinal studies, prospective studies, and RCTs. Longitudinal studies that examine the temporal variability and potential progression of brain fog and its effects on brain morphology, connectivity, cognitive processing, and quality of life have been called for.^36,43,56,58,82,90,93^ Next, prospective cohort studies will provide insight into the onset of brain fog, because currently there is no clear explanation.^89,93^ Last, RCTs are necessary to evaluate the quality and efficacy of proposed interventions.^92^

Regardless of the type of study, future research should also include both subjective and objective neuropsychological measures. Well-validated subjective measures are important to understand patient experiences; however, some studies have posited that they may be insufficient.^79^

Objective measures are important to support precise statistical modeling; however, neuropsychological measures are time- and resource-consuming and do not capture the holistic experience of chronic pain.^46^ The incorporation of both types of assessments, when possible, can counterbalance each assessment’s limitations.^10,46,68,79,82,84,87,93^

### Limitations

The findings of this review should be weighed with consideration of its limitations. The first obstacle encountered was that brain fog in chronic pain was ill defined and was not explicitly used in the literature, with the exception of one study. Thus, we were reliant on our pre-established definition based on a thematic summary of a nonexhaustive literature search, which was vetted by patient and health care partners. We chose to construct our own definition relative to chronic pain rather than rely on definitions from similar populations because of the variability of brain fog origins and symptoms in different disorders.^10–12^ Though our established definition is similar to existing definitions of brain fog in other conditions, we have uniquely emphasized that brain fog is a heterogenous phenomenon leading to limitations for participation in functional activities. Other definitions have instead focused solely on cognitive impairments. For example, chemo fog was defined as “differences in issues with memory, attention, processing speed, and executive function.”^88(p1345)(P1345)^ Next, in chronic fatigue syndrome it has been defined as “slow thinking, difficulty focusing, confusion, lack of concentration, forgetfulness, or a haziness of thought process.”^94(p1)^ Thirdly, in neuropsychiatric diseases (e.g., schizophrenia), brain fog has been described as “reduced cognition, inability to concentrate and multitask, as well as loss of short term and long-term memory.”^9(p1)(P1)^ This limitation also speaks to the pressing need for a consistent definition of brain fog that can be applied in research and health care settings. A second limitation is that we used the construct of cognitive dysfunction to help guide our search. However, as noted in our discussion, the findings from the literature demonstrate that this term also has a broad scope, which may have influenced the relevance of our findings. A third limitation is that the search was limited to studies published in English, which introduces a potential language bias. Additionally, although medication is a potential confounder in brain function, it was not possible to exclude sources that included participants who used medication, because the majority of persons with chronic pain typically rely on a form of medication to manage their pain. Therefore, the possibility of brain fog in chronic pain being merely a symptom of pain medication cannot be excluded. However, given the prevalence of brain fog in other conditions (e.g., depression,^7^ long COVID,^8^ and chronic fatigue syndrome^10^), it is likely to be linked to numerous causal factors. Lastly, we deviated from our protocol and did not include gray literature as was originally intended. Because many authors suggested that brain fog and fibro-fog are the common terminology used by patients,^44,49–51^ excluding gray literature may have missed important lay discourses and lived experience insights on this phenomenon.

#### Recommendations for Future Research

This review has demonstrated the urgent need for a coherent definition of brain fog. As such, we propose that a Delphi study investigating the differences in how patients and HCPs perceive and define brain fog should be conducted. Delphi studies are used to obtain consensus on a construct from a group of experts. Comparing lived experiences with HCPs’ perceptions will help us understand identify any gaps in understanding between these stakeholder groups. Importantly, using lived patient experiences will help us understand which areas of research are most important to patients’ overall quality of life.

### Clinical Implications

In recent years there has been considerable focus on cognitive-based strategies for the management of chronic pain, including pain neuroscience education,^95,96^ cognitive behavioral therapy, and mindfulness training.^96–98^ These interventions require sufficient cognitive resources to support success and, as such, may have limited efficacy in persons with brain fog who may not have the cognitive capacity to engage with the treatment.^26^ To obtain an understanding of how brain fog may be affecting treatment response and engagement, it needs to be included as a modifiable outcome and/or effect modifier in studies. This will provide an understanding of how brain fog can potentially be affected by interventions or how it could affect other outcomes such as patient satisfaction, quality of life, and sleep. However, for brain fog to be included as a modifiable outcome in research studies, it requires a consistent definition.

## Conclusion

This review collated the notable challenges produced by brain fog in persons with chronic pain, current literature gaps, the constraints of past research, and evidence-based suggestions for future research. These factors emphasize the pressing need for research to systematically address the evaluation and treatment of the effects of brain fog in persons with chronic pain. Through a collaboration with HCPs and patient partners, we proposed a model and definition rooted in the existing evidence.

Currently, there are only measures for surrogates of brain fog but not for the actual multidimensional subjective experience of this phenomenon. We are calling for the development of specific measures to comprehensively address the effect of brain fog on one’s ability to meaningfully engage in their daily living. This development may benefit from qualitative research that investigates the lived experiences of persons with chronic pain and brain fog. The provided model of the contributors of brain fog (Figure 3) and proposed definition can be used to guide researchers as they investigate robust and relevant assessments and clinical interventions to address brain fog. It may also help in generating hypotheses to test when modeling risks and predictors of outcomes and inform subgroup analysis of patient characteristics, ensuring consideration of important variables. To summarize, there is an urgent need for earnest academic and clinical consideration of brain fog in chronic pain. The current inconsistency in research regarding brain fog in chronic pain is obstructing a clear understanding of the phenomenon and therefore may be impeding persons with chronic pain and brain fog from receiving optimal care.

## Appendices Appendix A.

Category 1: Chronic pain

Category 2: Brain Fog

1. Brain Fog

2. Cognitive Impairment/ dysfunction/ dysregulation Category

3: Cognitive abilities

1. Mental competency/ processes

2. Memory

3. Perception

4. Emotion cognition

5. Attention

6. Critical thinking

7. Executive function

## Appendix B. Paper information

**Table:**

Study	Country	Study design	Level of evidence	Sample size	Purpose	Brain fog defined (Y/N)
Eccleston^54^	England	Repeated measures between subjects	3	CP = 22	The experience of pain’s impact on attentional control tasks	N
Landrø et al.^57^	Norway	Cross-sectional	3	FM = 25 HC = 18	Compare the memory functioning of patients with FM to HC	N
Schnurr^73^	England	Cross-sectional	3	CP = 134	Investigate memory complaints in CP participants	N
Iezzi et al.^81^	England	Cross-sectional	3	CP = 73	Investigate the relationship between chronic pain and attention	N
Koutantji et al.^56^	England	Quasi-experimental mixed model	3	High pain = 18 Low pain = 18	Compare the processing of pain related words in participants with high vs. low pain	N
McCracken and Iverson^65^	United States	Cross-sectional	3	CP = 275	Examined predictors of cognitive complaints in participants with CP	N
Witty et al.^84^	United States	Cross-sectional	3	CP = 78	Examined problem solving ability and pain ratings in participants with CP	N
Dick et al.^53^	Canada	Cross-sectional	3	FM = 20 RA = 20 MSK = 20 HC = 20	Compare attentional functioning in FM vs. RA vs. MSK vs. HC	N
Sephton et al.^72^	United States	Cross-sectional	3	FM = 50	Explore relationship between memory function and biological/psychological factors in participants with FM	N
Apkarian et al.^52^	United States	Cross-sectional	3	CP = 26 HC = 26 CRPS = 12	Investigate emotional decision making in CP vs. HC vs. CRPS	N
Roth et al.^83^	United States	Cross-sectional	3	CP = 222	Explore relationship between external factors and cognitive dysfunction in participants with CP	N
Veldhuijzen et al.^99^	United States	Cross-sectional	3	CP = 14 HC = 14	Explore the effects of pain on driving performance	N
Ling et al.^89^	England	Independent group design	3	CP = 72	Experience memory deficits in participants with CP	N
Berg et al.^80^	United States	Quasi-experimental	2	CP = 60	Examine relationship between pain intensity and concentration	N
Dick et al.^78^	Canada	Cross-sectional	3	N/A	Examine mechanisms of cognitive disruption in FM	N
Lee et al.^58^	England	Cohort	3	HC = 1273 CWP = 266	Explore association between CWP and cognition	N
Glass et al.^43^	United States	Cross-sectional	3	FM = 18 HC = 14	Explored executive function in participants with FM	N
Reyes Del Paso et al.^75^	Spain	Cross-sectional	3	FM = 35	Evaluate cognitive performance in participants with FM	N
Pulles and Oosterman^63^	Netherlands	Cross-sectional	3	CP = 30	Explore the relationship between pain intensity and neuropsychological function in participants with CP	N
Williams et al.^50^	United States	Cross-sectional	3	FM = 72	Explore types of cognitive dysfunction in participants with FM	Y
Seo et al.^59^	Spain	Cross-sectional	2	FM = 19 HC = 22	Investigate differences in neural correlates of working memory in FM vs. HC	N
Landrø et al.^79^	Norway	Cross-sectional	3	CP = 72	Explore neuropsychological functioning in participants with pain	N
Isbir et al.^74^	Turkey	Cross-sectional	3	CP = 98	Explore relationship between chronic pain and cognitive function	N
Martinsen et al.^46^	Sweden	Cohort	2	FM = 29 HC = 31	Explore differences in cognitive function in FM vs. HC	Y
Shmygalev et al.^76^	Germany	Cross-sectional	3	FM = 43 HC = 129	Assess driving performance in FM vs. HC	N
Coppieters et al.^62^	Belgium	Case-control	3	CINP = 35 CWAD = 32 HC = 28	Examine differences in cognitive performance in CINP vs. CWAD vs. HC	N
Tamburin^100^	Italy	Cross-sectional	3	CLBP = 24 HC = 24	Explore cognitive performance of participants with CLBP	N
Grisart and Plaghki^64^	United States	Cross-sectional	3	CLBP = 17 FM = 16	Explore mechanisms contributing to cognitive impairment	N
Coppieters et al.^61^	Belgium	Case control	4	CP = 28 CIP = 35 CWAD = 32	Investigate cognitive impairment in CP vs. CIP vs. CWAD	N
Elvemo et al.^77^	Norway	Cross-sectional	3	CP = 20 HC = 20	Explore differences in working memory in participants with CP vs. HC	N
Kalfon et al.^45^	Israel	Cross-sectional	3	CP = 50	Explore cognitive impairment in participants with CP	Y
Ferreira et al.^101^	Brazil	Cross-sectional	3	CP = 45 HC = 45	Explore cognitive functioning in participants with CP vs. HC	N
Gunnarsson et al.^102^	Sweden	Cross-sectional	3	MSK = 214	Explore cognitive functioning in participants with MSK	N
Nadar et al.^93^	Kuwait	Cross-sectional	3	CP = 40 HC = 29	Explore cognitive functioning in participants with CP vs. HC	N
Ojeda et al.^85^	Spain	Cross-sectional	3	CP = 336	Investigate the validity of memory tool in participants with CP	N
Baker et al.^68^	Australia	Cross-sectional	3	*N* = 41	Explore relationship between self-report and objective pain measures	N
González-Villar et al.^55^	United States	Cross-sectional	3	FM = 35 HC = 35	Compare working memory in FM vs. HC	N
Lenoir et al.^90^	Belgium	Case-control	4	CWAD = 13 CIP = 18 FM = 33 HC = 33	Identify validity of tool in CWAD vs. participants with FM vs. HC	N
Ren et al.^66^	China	Cross-sectional	3	CP = 24 HC = 24	Explore cognitive functioning in participants with CP vs. HC	N
Schrier et al.^103^	Netherlands	Cross-sectional	3	CP = 287	Explore cognitive functioning in participants with CP	N
Verim et al.^104^	United States	Cross-sectional	3	CP = 55 HC = 40	Explore relationship between enolase levels and cognitive functioning in participants with CP vs. HC	N
Galvez Sánchez et al.^42^	Italy	Cross-sectional	3	FM = 42	Explored the relationship with cognition and affect in participants with FM	N
Gunendi et al.^44^	United States	Cross-sectional	3	FM = 15	Evaluate sensory processing in participants with FM	Y
Ojeda et al.^82^	Spain	Cross-sectional	3	MSK = 245 HC = 72	Assess cognitive performance in participants with MSK vs. HC	N
Taylor et al.^51^	England	Cross-sectional	3	CP = 941	Patient perspective of nonpharmacological vs. pharmacological treatments in participants with CP	N
Blanco et al.^40^	United States	Cross-sectional	3	FM = 146 HC = 122	Analyze cognitive and olfactory functioning in participants with FM vs. HC	N
Bothelius et al.^41^	Sweden	Cross-sectional	3	CP = 22	Assess neuropsychological function in participants with CP	N
Moore et al.^39^	United States	Cross-sectional	3	FM = 24 HC = 26	Assess cognitive functioning in participants with FM vs. HC	N
Whibley et al.^48^	United States	Cross-sectional	3	FM = 50 HC = 50	Investigate association between pain intensity and measures of cognitive function	N
Castel et al.^60^	Spain	Cross-sectional	3	FM = 70 CP = 74 HC = 40	Explore relationship of cognitive performance in FM vs. CP vs. HC	N
Moreira and Novak^35^	Czech Republic	RCT	2	CP = 40	Investigate whether pain affects cognition and mobility	N
Jacobsen et al.^34^	Norway	Randomized controlled crossover trial	2	CP = 73	Investigate the utility of MINDFlex	N
Samartin-Veiga et al.^69^	Spain	Cross-sectional	3	FM = 19 HC = 22	Identify relationship brain electrical activity and cognition in participants with FM vs. HC	N
Corti et al.^36^	Australia	Cross-sectional	3	FM = 34 HC = 30	Explore cognitive profile of participants with FM with cognitive impairments	N
Munoz and Esteve^71^	United States	Cross-sectional	3	CP = 149	Explore memory complaints in CP	N
Lupu et al.^92^	Israel	Cross-sectional	3	CP = 33 HC = 31	Use Cogstate Brief Battery to assess cognition in participants with CP	N
Pappolla et al.^49^	United States	Cross-sectional	3	FM = 13	Explore association between FM and insulin resistance	Y
Seward et al.^38^	United States	Cross-sectional	3	CLBP = 307	Characterize driving experience of participants with CLBP	N
Tiwari et al.^105^	India	Cross-sectional	3	FM = 34 HC = 30	Compare measures of cognition in FM vs. HC	N
Baker et al.^33^	United States	RCT	3	CP = 39	Examine the efficacy of computerized training for cognitive impairment in CP	N
Oosterman et al.^106,107^	Netheralands	Cross-sectional	3	CP = 34 HC = 32	Examine differences in executive and attentional control in CP vs. HC	N
Zhang et al.^29^	United States	Cross-sectional	3	CP = 20 HC = 25	Quantify differences in decision making in CP vs. HC	N
Liu et al.^91^	United States	Cross-sectional	3	CP = 331 HC = 333	Explore working and autobiographical memory in CP	N
Jorge et al.^70^	United States	Cross-sectional	3	RA = 33 CP = 24	Analyze memory deficits in CP	N
Russell et al.^24^	Germany	Qualitative (focus group)	5	FM = 14	Explore perceptions of exercise, sleep, and cognition	N
Gubler et al.^86^	Switzerland	Report study	5	CP = 33 HC = 33	Compare involuntary and voluntary attention in CP vs. HC	N
Berryman et al.^25^	Australia	Meta-analysis	1	CP	Explored working memory deficits in CP	N
Berryman et al.^26^	Australia	Meta-analysis	1	CP	Explored executive function deficits in CP	N
Bell et al.^27^	United States	Meta-analysis	1	CP	Synthesis of cognitive performance across FM studies	N
Innes and Sambamoorthi^28^	United States	Systematic review	1	CP	Evaluate association between CP and cognitive functioning	N
Mendonca et al.^47^	United States	Systematic review	1	FM	Investigate executive function in FM	N
Liu et al.^31^	Australia	Review	5	CP	Review of memory impairment factors in CP	N
Mazza et al.^32^	France	Review	5	CP	Review of memory impairments in CP	N
Glass^30^	United States	Review	5	CP	Explanation of dyscognition in FM	N
Galvez Sánchez et al.^67^	Italy	Cross-sectional	3	FM = 42	Explored the relationship with cognition and affect in participants with FM	N

Level of evidence defined by the Centre for Evidence-Based Medicine.

HC = healthy control; FM = fibromyalgia; RA = rheumatoid arthritis; MSK = musculoskeletal pain; CRPS = complex regional pain syndrome; CIP = chronic idiopathic disorder; CWAD = chronic whiplash-associated disorder; CP = chronic pain.

## Appendix C. Cognitive impairments

**Table:**

Category	Test	Study
Driving	Driving Behavior Questionnaire	Seward et al.^38^
	Driving Habits Questionnaire	Seward et al.^38^
	Driving Test Battery	Shmygalev et al.^76^
Memory	Interference Memory Test	Tamburin^100^
	Memory Observation Questionnaire	Schnurr^73^
	Test Your Memory	Ojeda et al.^82,85^
	Memory Task	Koutantji et al.^56^
	Everyday Memory Questionnaire	Baker et al.,^33,68^ Landrø et al.^79^
	Prospective Memory Questionnaire	Ling et al.^89^
	Test of Memory Malingering	Kalfon et al.^45^
	Incidental Memory	Landrø et al.^57^
	Auto evaluation of memory	Munoz and Esteve^71^
	Weschler Memory Scale	Schiltenwolf et al.,^37^ Blanco et al.,^40^ Apkarian et al.,^52^ Landrø et al.,^57,79^ Jorge et al.,^70^ Sephton et al.,^72^ Elvemo et al.,^77^ Iezzi et al.^81^
	Autobiographical Memory	Liu et al.^91^
	Memory Failures of Everyday	Castel et al.,^60^ Samartin-Veiga et al.^69^
	Rey-Osterrieth Figure	Galvez-Sánchez et al.,^42,67^ Lee et al.,^58^ Iezzi et al.,^81^ Lenoir et al.^90^
	Pattern Recognition Memory	Schiltenwolf et al.^37^
	Rivermead Behavioral Memory Test–Story Recall	Pulles and Oosterman^63^
	Dot Memory Test	Whibley et al.^48^
	Category fluency	Pulles and Oosterman^63^
	Paired associates learning	Jacobsen et al.^34^
	Stroop task	Baker et al.,^33,68^ Apkarian et al.,^52^ Pulles and Oosterman,^63^ Coppieters et al.,^61,62^ Iezzi et al.,^81^ Ferreira et al.^101^
		Castel et al.,^60^ Lenoir et al.^90^
	Reading Span task	Dick et al.^78^
	Working Memory Index	Liu et al.^31,91^
	Number sequencing	Corti et al.,^36^ Elvemo et al.^77^
	Spatial Working Memory	Jacobsen et al.^34^
Language	Controlled Oral Word Association	Iezzi et al.^81^
	Language Boston Naming	Corti et al.^36^
	Multiple Choice Vocabulary	Corti et al.^36^
	Matrix reasoning and vocabulary	Landrø et al.^79^
	Verbal learning	Corti et al.,^36^ Galvez-Sánchez et al.,^42,67^ Castel et al.,^60^ Landrø et al.^79^
Executive Function	Card Sorting Task	Baker et al.,^33,68^ Jacobsen et al.,^34^ Apkarian et al.,^52^ Iezzi et al.,^81^ Tamburin^100^
	Design Fluency	Iezzi et al.^81^
	Zoo Mapping Task	Coppieters et al.,^62^ Pulles and Oosterman,^63^ Galvez-Sánchez et al.^67^
	Revised Strategy Application	Galvez-Sánchez et al.^67^
	Behavior Rating Inventory of Executive Function	Baker et al.^68^
	Cambridge Neuropsychological Test	Corti et al.,^36^ Schiltenwolf et al.^37^
	Iowa Gambling	Zhang et al.,^29^ Apkarian et al.,^52^ Elvemo et al.,^77^ Tamburin^100^
	Paced Auditory Serial Addition	Iezzi et al.^81^
	Problem Solving Confidence	Witty et al.^84^
	Go/no go	Elvemo et al.^77^
	Stop signal task	Jacobsen et al.^34^
Attention	Trail Making	Baker et al.,^33,68^ Schiltenwolf et al.,^37^ Galvez-Sánchez et al.,^42^ Coppieters et al.,^62^ Pulles and Oosterman,^63^ Iezzi et al.,^81^ Tamburin,^100^ Nadar et al.^93^
	Spatial Span	Schiltenwolf et al.^37^
	Complex Concentration Task	Berg et al.^80^
	Bourdon Vos Test	Coppieters et al.,^62^ Pulles and Oosterman^63^
	Oddball Task	Gubler et al.^86^
	Toulouse-Piéron Perceptual and Attention Test	Castel et al.^60^
	Attention Switching Task	Jacobsen et al.^34^
	Symbol Digit Modalities Test	Baker et al.^68^
	Test of Everyday Attention	Dick et al.^78^
General Cognitive Functioning	Multiple Abilities Self Report	Williams et al.^50^
	Digital Span Task	Apkarian et al.,^52^ Landrø et al.,^57^ Pulles and Oosterman,^63^ Elvemo et al.,^77^ Dick et al.,^78^ Tamburin^100^
	Cognitive Failure Questionnaire	Baker et al.,^33,^ Schier^103^
	Contextual Memory task	Nadar et al.^93^
	IQ tests	Schiltenwolf et al.,^37^ Apkarian et al.,^52^ Landrø et al.,^57,79^ Sephton et al.,^72^ Elvemo et al.,^77^ Iezzi et al.^81^
	National Adult Reading Test	Pulles and Oosterman^63^
Screening	Montreal Cognition Assessment	Isbir et al.,^74^ Ferreira et al.^101^
	Mini-Mental State	Pulles and Oosterman,^63^ Ojeda et al.,^85^ Tiwari et al.^105^
Other	Visuospatial Judgment of Line Orientation	Corti et al.^36^
	Motor Coordination	Shmygalev et al.^76^
	Vigilance	Coppieters et al.,^61^ Shmygalev et al.^76^
	CANTAB	Jacobsen et al.,^34^ Corti et al.,^36^ Schiltenwolf et al.^37^
	Symbol Search test	Whibley et al.^48^
	Perceived Deficits Questionnaire	Coppieters et al.^62^

## Appendix D. Participation in daily activities and quality of life impairments

**Table:**

Category	Subcategory	Test	Paper
Emotional Functioning		Pain Catastrophizing Scale	Baker et al.,^33^ Seward et al.,^38^ Pulles and Oosterman,^63^ Grisart and Plaghki,^64^ Munoz and Esteve^71^
		Positive and Negative Affect Scale	Galvez-Sánchez et al.^42^
		Toronto Alexithymia Scale	Galvez-Sánchez et al.^42^
		Coping Strategies Questionnaire	Castel et al.,^60^ Jorge et al.,^70^ Roth et al.^83^
		Connor-Davidson Resilience Scale	Schier^103^
		Perceived Stress Scale	Sephton et al.^72^
Mental Health	Depression Anxiety Other	Beck Depression Inventory	Kalfon et al.,^45^ González-Villar et al.,^55^ Landrø et al.,^57^ Galvez Sanchez et al.,^67^ Baker et al.,^68^ Shmygalev et al.,^76^ Roth et al.^83^
		Hospital Anxiety and Depression Scale	Martinsen et al.,^46^ Eccleston,^54^ Castel et al.,^60^ Grisart and Plaghki,^64^ Jorge et al.,^70^ Munoz and Esteve,^71^ Dick et al.,^78^ Gubler et al.,^86^ Shrier^103^
		Zung Depression Scale	Ling et al.^89^
		State-Trait Anxiety Inventory	Galvez-Sanchez et al.,^42^ Martinsen et al.,^46^ Shmygalev et al.^76^
		PCPT Spell It Out	Roth et al.^83^
		Pain Anxiety Symptoms Scale	Grisart and Plaghki^64^
Pain experiences and impairment		Pain Experience Scale	Berg et al.^80^
		Pain Disability Index	Berg et al.^80^
		Visual Analogue Scale	Jorge et al.,^70^ Gubler et al.,^86^ Liu et al.^91^
		Neck Pain-Related Disability	Coppieters et al.^61^
		Numerical Pain Intensity Rating Scale	Castel et al.,^60^ Grisart and Plaghki^64^
		Brief Pain Inventory	Zhang et al.,^29^ Baker et al.,^33,68^ Shmygalev et al.,^76^ Lupu et al.^92^
		Pain Rating Index	Kalfon et al.,^45^ Martinsen et al.,^46^ González-Villar et al.,^55^ Pulles and Oosterman,^63^ Shmygalev et al.,^76^ Nadar et al.^93^
			Schiltenwolf et al.,^37^ Whibley et al.,^48^ Baker et al.^68^
		Fibromyalgia Survey Questionnaire	González-Villar et al.,^55^ Samartin-Viega et al.^69^
		McGill Pain Questionnaire	Galvez-Sanchez et al.,^42^ Dick et al.,^78^ Roth et al.^83^
		Pain Self-Efficacy Questionnaire	Baker et al.^33^
General measures of QOL		Sickness Impact Profile	McCracken and Iverson,^65^ Witty et al.^84^
		Roland Morris Disability Questionnaire	Corti et al.^36^
		Patient Health Questionnaire	Mackay^7^
		Disability Rating Index	Pulles and Oosterman^63^
		SF-36	Martinsen et al.,^46^ Coppieters et al.,^61^ Pulles and Oosterman,^63^ Dick et al.,^78^ Ojeda et al.^85^
